# 5-Meth­oxy-2-[(5-meth­oxy-1*H*-indol-1-yl)carbon­yl]-1*H*-indole

**DOI:** 10.1107/S1600536812020399

**Published:** 2012-05-19

**Authors:** Mohamed I. Attia, Nasser R. El-Brollosy, Ali A. El-Emam, Seik Weng Ng, Edward R. T. Tiekink

**Affiliations:** a Department of Pharmaceutical Chemistry, College of Pharmacy, King Saud University, Riyadh 11451, Saudi Arabia; b Department of Chemistry, University of Malaya, 50603 Kuala Lumpur, Malaysia; c Chemistry Department, Faculty of Science, King Abdulaziz University, PO Box 80203 Jeddah, Saudi Arabia

## Abstract

The asymmetric unit of the title compound, C_19_H_16_N_2_O_3_, comprises three independent mol­ecules (*A*, *B* and *C*). The inversion-related molecule of *A* is virtually superimposable upon the other two molecules. In each mol­ecule, there is a twist in the link between the approximately *syn* carbonyl and amine groups [the N—C—C—O torsion angles range from 19.73 (19) to −21.2 (2)°]. Each mol­ecule has a bent shape quanti­fied in terms of the dihedral angle between the indole and indole fused-ring systems [range = 45.69 (5)–47.91 (5)°]. In the crystal, the *A* and *B* mol­ecules form dimeric aggregates *via* ten-membered {⋯HNC_2_O}_2_ synthons, while the *C* mol­ecules self-associate similarly but about a centre of inversion.

## Related literature

For background to melatonin and melatonin preparations, see: Barrenetxe *et al.* (2004 ▶); Williamson *et al.* (1998 ▶). For background to melatonin receptor ligands, see: Bedini *et al.* (2006 ▶); Attia *et al.* (2008 ▶). For a related structure, see: Attia *et al.* (2012 ▶).
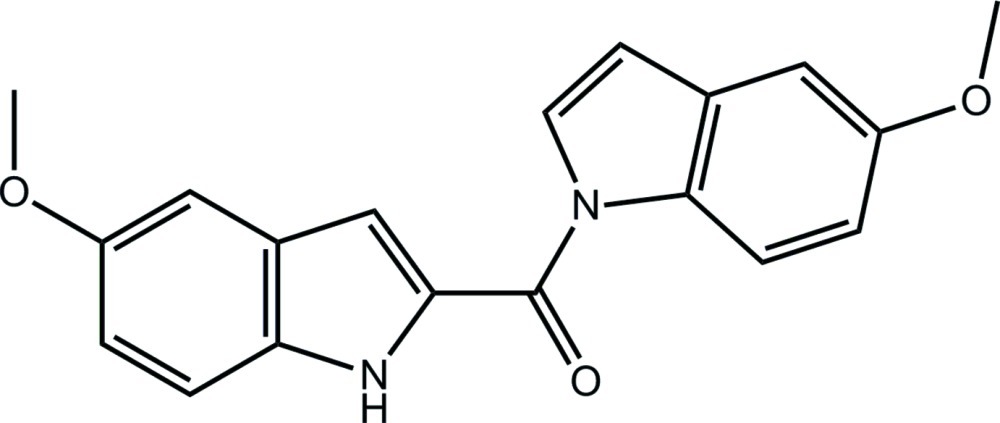


## Experimental

### 

#### Crystal data

C_19_H_16_N_2_O_3_
*M* *_r_* = 320.34
Triclinic, 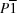

*a* = 11.3153 (4) Å
*b* = 12.1183 (5) Å
*c* = 17.1300 (6) Å
α = 76.251 (3)°
β = 79.747 (3)°
γ = 88.913 (3)°
*V* = 2244.50 (15) Å^3^
*Z* = 6
Cu *K*α radiation
μ = 0.80 mm^−1^
*T* = 100 K
0.30 × 0.20 × 0.02 mm

#### Data collection

Agilent SuperNova Dual diffractometer with an Atlas detector
Absorption correction: multi-scan (*CrysAlis PRO*; Agilent, 2011 ▶) *T*_min_ = 0.367, *T*_max_ = 1.000
17372 measured reflections
9182 independent reflections
7534 reflections with *I* > 2σ(*I*)
*R*_int_ = 0.029

#### Refinement

*R*[*F* ^2^ > 2σ(*F* ^2^)] = 0.043
*wR*(*F* ^2^) = 0.121
*S* = 1.02
9182 reflections
661 parameters
H atoms treated by a mixture of independent and constrained refinement
Δρ_max_ = 0.28 e Å^−3^
Δρ_min_ = −0.30 e Å^−3^

### 

Data collection: *CrysAlis PRO* (Agilent, 2011 ▶); cell refinement: *CrysAlis PRO*; data reduction: *CrysAlis PRO*; program(s) used to solve structure: *SHELXS97* (Sheldrick, 2008 ▶); program(s) used to refine structure: *SHELXL97* (Sheldrick, 2008 ▶); molecular graphics: *ORTEP-3* (Farrugia, 1997 ▶), *Qmol* (Gans & Shalloway, 2001 ▶) and *DIAMOND* (Brandenburg, 2006 ▶); software used to prepare material for publication: *publCIF* (Westrip, 2010 ▶).

## Supplementary Material

Crystal structure: contains datablock(s) global, I. DOI: 10.1107/S1600536812020399/su2424sup1.cif

Structure factors: contains datablock(s) I. DOI: 10.1107/S1600536812020399/su2424Isup2.hkl

Supplementary material file. DOI: 10.1107/S1600536812020399/su2424Isup3.cml

Additional supplementary materials:  crystallographic information; 3D view; checkCIF report

## Figures and Tables

**Table 1:** Hydrogen-bond geometry (Å, °)

*D*—H⋯*A*	*D*—H	H⋯*A*	*D*⋯*A*	*D*—H⋯*A*
N1—H1*n*⋯O5	0.91 (2)	1.98 (2)	2.8536 (16)	159 (2)
N3—H3*n*⋯O2	0.91 (2)	1.97 (2)	2.8384 (16)	159.0 (19)
N5—H5*n*⋯O8^i^	0.90 (2)	2.00 (2)	2.8796 (15)	163.9 (19)

Symmetry code: (i) 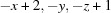.

**Fig. 1.**
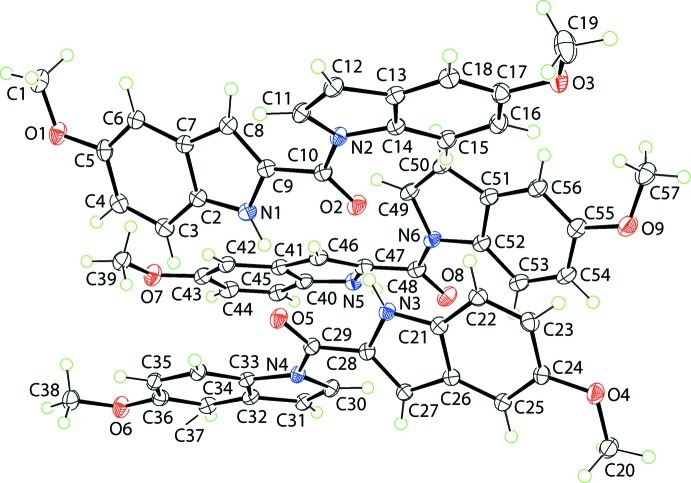
The molecular structure of the title compound (I), showing the atom-labelling and displacement ellipsoids drawn at the 50% probability level.

**Fig. 2.**
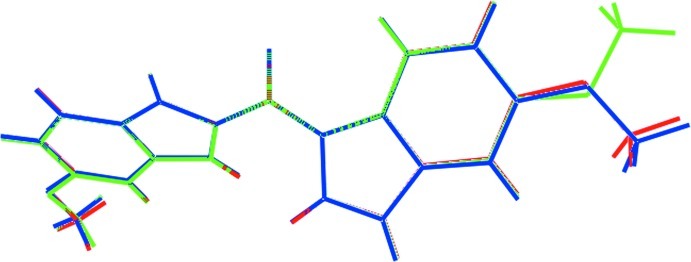
Overlay diagram of the N1- (red), N3- (green) and N5- (blue) containing molecules in (I) aligned so that the central amide residues are coincident.

**Fig. 3.**
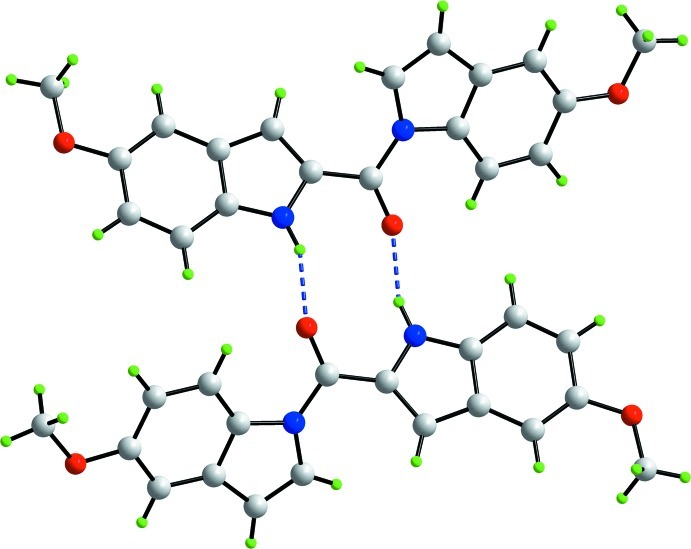
A view of a dimeric aggregate in (I) sustained by N—H···O hydrogen bonds, shown as blue dashed lines.

## supplementary crystallographic information

### Comment

Melatonin (*N*-acetyl-5-methoxytryptamine, MLT) is primarily produced by the pineal gland in the brain with a marked circadian rhythm normally peaking in the dark to regulate sleep patterns (Barrenetxe *et al.*, 2004). It has been reported that commercial melatonin preparations contain *N*-{2-[1-({3-[2-(acetylamino)ethyl]-5-methoxy-1*H*-indol-2-yl}-methyl)-5-methoxy-1*H*-indol-3-yl]ethyl}acetamide (1) as a contaminant (Williamson *et al.*, 1998). The title compound, namely (5-methoxy-1*H*-indol-1-yl)(5-methoxy-1*H*-indol-2-yl)methanone (I), can be elaborated to give compound 1, in four steps. The synthesis of compound 1 on a preparative scale is required for the development of an analytical method for the determination of MLT in the presence of this contaminant in commercial MLT preparations. Herein, the crystal and molecular structure of the title compound (I) is described in continuation of on-going studies of melatonin receptor ligands (Bedini *et al.*, 2006; Attia *et al.*, 2008; Attia *et al.*, 2012).

Three crystallographically independent molecules comprises the asymmetric unit of the title compound (I), Fig. 1. In each molecule there is a twist in the link between the carbonyl and amine groups but, each of these is approximately *syn* with the N1—C9—C10—O2, N3—C28—C29—O5 and N5—C47—C48—O8 torsion angles being 19.73 (19), -20.34 (19) and -21.2 (2)°, respectively. Each molecule has a bent shape quantified in terms of the dihedral angle between the indole and indonyl fused ring systems. For the N1-containing molecule this angle is 45.69 (5)° which compares to 45.86 (5) and 47.91 (5)° in the other two molecules. If the inversion-related N1-containing molecule is overlapped with the N2- and N3-containing molecules, it can be seen that all three molecules are virtually superimposable, as shown in Fig. 2. The major differences are apparent in the relative orientations of the terminal methoxy groups of the indonyl rings. In the N1- and N-3 containing molecules, the methyl group is orientated in almost the opposite direction to that seen in the N2-containing molecule. Further, in the N3-containing molecule, the methoxy group is slightly twisted out of the plane of the benzene ring to which it is connected. This is quantified in the values of the C16—C17—O3—C19, C35—C36—O6—C37 and C54—C55—O9—C57 torsion angles of -179.18 (14), 0.06 (2) and 168.68 (13)°, respectively.

In the crystal, the N1- and N2-containing molecules associate *via N*—*H*···*O*(carbonyl) hydrogen bonds to form dimeric aggregates *via* 10-membered {···HNC_2_O}_2_ synthons, Fig. 3. The N3-containing molecules self-associate similarly but about a centre of inversion. Molecules assemble into a three-dimensional architecture *via*π—π interactions with the closest of these occurring between five-membered (N2,C11–C14) and six-membered C32–C37 rings [inter-centroid distance = 3.5307 (9) Å for symmetry operation *(i)* = -*x+1*, -*y+1*, -*z+1*].

### Experimental

A mixture of (5-methoxy-2,3-dihydro-1*H*-indol-1-yl)(5-methoxy-1*H*-indol-2-yl)- methanone (0.20 g, 0.62 mmol) and 2,3-dichloro-5,6-dicyanobenzoquinone (0.19 g, 0.68 mmol) in ethyl acetate (30 ml) was heated at reflux temperature for 18 h. The reaction mixture was evaporated under reduced pressure and the residue was purified by silica gel chromatography (chloroform/methanol/ammonia, 10:1:0.1) to furnish 0.19 g (96%) of (5-methoxy-1*H*-indol-1-yl)(5-methoxy-1*H*-indol-2-yl)methanone as a light-red powder which was recrystallized from ethanol to give colourless crystals of the title compound (I); *M*.pt: 451–452 K.

### Refinement

Carbon-bound H-atoms were placed in calculated positions [C—H = 0.95 to 0.98 Å, *U*_iso_(H) = 1.2*U*_eq_(C)] and were included in the refinement in the riding model approximation. The amino H-atoms were refined freely. The (5 11 17) reflection was omitted owing to poor agreement.

### Crystal data

**Table:**

C_19_H_16_N_2_O_3_	*Z* = 6
*M**_r_* = 320.34	*F*(000) = 1008
Triclinic, *P*1	*D*_x_ = 1.422 Mg m^−^^3^
Hall symbol: -P 1	Cu *K*α radiation, λ = 1.54184 Å
*a* = 11.3153 (4) Å	Cell parameters from 7164 reflections
*b* = 12.1183 (5) Å	θ = 2.7–76.2°
*c* = 17.1300 (6) Å	µ = 0.80 mm^−^^1^
α = 76.251 (3)°	*T* = 100 K
β = 79.747 (3)°	Plate, colourless
γ = 88.913 (3)°	0.30 × 0.20 × 0.02 mm
*V* = 2244.50 (15) Å^3^	

### Data collection

**Table:**

Agilent SuperNova Dual diffractometer with an Atlas detector	9182 independent reflections
Radiation source: SuperNova (Mo) X-ray Source	7534 reflections with *I* > 2σ(*I*)
Mirror monochromator	*R*_int_ = 0.029
Detector resolution: 10.4041 pixels mm^-1^	θ_max_ = 76.4°, θ_min_ = 2.7°
ω scan	*h* = −14→13
Absorption correction: multi-scan (*CrysAlis PRO*; Agilent, 2011)	*k* = −10→15
*T*_min_ = 0.367, *T*_max_ = 1.000	*l* = −21→21
17372 measured reflections	

### Refinement

**Table:**

Refinement on *F*^2^	Primary atom site location: structure-invariant direct methods
Least-squares matrix: full	Secondary atom site location: difference Fourier map
*R*[*F*^2^ > 2σ(*F*^2^)] = 0.043	Hydrogen site location: inferred from neighbouring sites
*wR*(*F*^2^) = 0.121	H atoms treated by a mixture of independent and constrained refinement
*S* = 1.02	*w* = 1/[σ^2^(*F*_o_^2^) + (0.0672*P*)^2^ + 0.3331*P*] where *P* = (*F*_o_^2^ + 2*F*_c_^2^)/3
9182 reflections	(Δ/σ)_max_ < 0.001
661 parameters	Δρ_max_ = 0.28 e Å^−^^3^
0 restraints	Δρ_min_ = −0.30 e Å^−^^3^

### Special details

**Table:**

Geometry. All e.s.d.'s (except the e.s.d. in the dihedral angle between two l.s. planes) are estimated using the full covariance matrix. The cell e.s.d.'s are taken into account individually in the estimation of e.s.d.'s in distances, angles and torsion angles; correlations between e.s.d.'s in cell parameters are only used when they are defined by crystal symmetry. An approximate (isotropic) treatment of cell e.s.d.'s is used for estimating e.s.d.'s involving l.s. planes.
Refinement. Refinement of *F*^2^ against ALL reflections. The weighted *R*-factor *wR* and goodness of fit *S* are based on *F*^2^, conventional *R*-factors *R* are based on *F*, with *F* set to zero for negative *F*^2^. The threshold expression of *F*^2^ > σ(*F*^2^) is used only for calculating *R*-factors(gt) *etc*. and is not relevant to the choice of reflections for refinement. *R*-factors based on *F*^2^ are statistically about twice as large as those based on *F*, and *R*- factors based on ALL data will be even larger.

### Fractional atomic coordinates and isotropic or equivalent isotropic displacement parameters (Å^2^)

**Table:**

	*x*	*y*	*z*	*U*_iso_*/*U*_eq_	
O1	0.87563 (11)	0.89787 (9)	0.11597 (6)	0.0252 (2)	
O2	0.71161 (10)	0.56194 (8)	0.59096 (7)	0.0228 (2)	
O3	0.72114 (11)	0.68238 (10)	0.93945 (7)	0.0270 (2)	
O4	0.45990 (10)	0.07927 (9)	0.91666 (6)	0.0247 (2)	
O5	0.59785 (10)	0.42667 (8)	0.44330 (6)	0.0220 (2)	
O6	0.62516 (11)	0.29566 (9)	0.09143 (6)	0.0261 (2)	
O7	0.92792 (11)	0.40367 (9)	0.10246 (7)	0.0279 (2)	
O8	0.91510 (10)	0.07096 (8)	0.57278 (7)	0.0252 (2)	
O9	0.81693 (11)	0.19031 (9)	0.92812 (7)	0.0278 (2)	
N1	0.71116 (11)	0.64193 (10)	0.42505 (8)	0.0187 (2)	
N2	0.71456 (10)	0.74048 (9)	0.61092 (7)	0.0173 (2)	
N3	0.60355 (11)	0.34591 (10)	0.60844 (7)	0.0178 (2)	
N4	0.60440 (10)	0.24863 (9)	0.42172 (7)	0.0165 (2)	
N5	0.97901 (11)	0.14977 (10)	0.40478 (8)	0.0210 (2)	
N6	0.91034 (11)	0.24988 (9)	0.59292 (8)	0.0192 (2)	
C1	0.92781 (15)	1.00741 (13)	0.10623 (9)	0.0258 (3)	
H1A	0.9528	1.0425	0.0479	0.039*	
H1B	0.9979	1.0000	0.1333	0.039*	
H1C	0.8686	1.0551	0.1308	0.039*	
C2	0.74608 (12)	0.69609 (11)	0.34483 (9)	0.0178 (3)	
C3	0.73470 (13)	0.66026 (12)	0.27422 (9)	0.0206 (3)	
H3A	0.6974	0.5894	0.2774	0.025*	
C4	0.78003 (13)	0.73241 (12)	0.20039 (9)	0.0214 (3)	
H4	0.7735	0.7108	0.1515	0.026*	
C5	0.83610 (13)	0.83791 (12)	0.19513 (9)	0.0208 (3)	
C6	0.84677 (12)	0.87432 (11)	0.26392 (9)	0.0183 (3)	
H6	0.8838	0.9456	0.2599	0.022*	
C7	0.80051 (12)	0.80150 (11)	0.34081 (9)	0.0174 (3)	
C8	0.79569 (12)	0.80978 (11)	0.42263 (9)	0.0176 (3)	
H8	0.8252	0.8714	0.4397	0.021*	
C9	0.73965 (12)	0.71070 (11)	0.47286 (9)	0.0174 (3)	
C10	0.72046 (12)	0.66481 (11)	0.56109 (9)	0.0175 (3)	
C11	0.67545 (12)	0.85333 (11)	0.59257 (9)	0.0187 (3)	
H11	0.6628	0.8947	0.5403	0.022*	
C12	0.65860 (13)	0.89380 (11)	0.66068 (9)	0.0193 (3)	
H12	0.6327	0.9677	0.6644	0.023*	
C13	0.68686 (12)	0.80492 (11)	0.72678 (9)	0.0180 (3)	
C14	0.72090 (12)	0.71065 (11)	0.69460 (9)	0.0171 (3)	
C15	0.75692 (12)	0.61024 (11)	0.74267 (9)	0.0194 (3)	
H15	0.7817	0.5472	0.7203	0.023*	
C16	0.75493 (13)	0.60653 (12)	0.82426 (9)	0.0213 (3)	
H16	0.7789	0.5394	0.8586	0.026*	
C17	0.71818 (13)	0.69985 (13)	0.85763 (9)	0.0210 (3)	
C18	0.68459 (13)	0.79993 (12)	0.80932 (9)	0.0204 (3)	
H18	0.6607	0.8632	0.8316	0.024*	
C19	0.68398 (19)	0.77366 (16)	0.97647 (11)	0.0368 (4)	
H19A	0.6901	0.7517	1.0345	0.055*	
H19B	0.6005	0.7915	0.9711	0.055*	
H19C	0.7357	0.8407	0.9493	0.055*	
C20	0.39862 (14)	−0.02612 (13)	0.92700 (9)	0.0248 (3)	
H20A	0.3792	−0.0634	0.9853	0.037*	
H20B	0.4501	−0.0751	0.8982	0.037*	
H20C	0.3243	−0.0124	0.9047	0.037*	
C21	0.57271 (12)	0.29019 (11)	0.68871 (9)	0.0172 (3)	
C22	0.58362 (13)	0.32549 (11)	0.75945 (9)	0.0189 (3)	
H22	0.6169	0.3981	0.7564	0.023*	
C23	0.54416 (13)	0.25078 (12)	0.83326 (9)	0.0204 (3)	
H23	0.5508	0.2721	0.8822	0.024*	
C24	0.49362 (13)	0.14248 (12)	0.83807 (9)	0.0187 (3)	
C25	0.48224 (12)	0.10716 (11)	0.76895 (8)	0.0175 (3)	
H25	0.4480	0.0347	0.7726	0.021*	
C26	0.52316 (12)	0.18238 (11)	0.69255 (8)	0.0166 (3)	
C27	0.52691 (12)	0.17516 (11)	0.61035 (8)	0.0169 (3)	
H27	0.4999	0.1127	0.5930	0.020*	
C28	0.57739 (12)	0.27657 (11)	0.56054 (8)	0.0166 (3)	
C29	0.59421 (12)	0.32370 (11)	0.47242 (8)	0.0172 (3)	
C30	0.64991 (13)	0.13806 (11)	0.43902 (9)	0.0191 (3)	
H30	0.6616	0.0964	0.4914	0.023*	
C31	0.67440 (12)	0.10014 (11)	0.36993 (9)	0.0191 (3)	
H31	0.7052	0.0279	0.3656	0.023*	
C32	0.64556 (12)	0.18867 (11)	0.30381 (9)	0.0176 (3)	
C33	0.60260 (12)	0.28048 (11)	0.33724 (8)	0.0163 (3)	
C34	0.56364 (12)	0.37893 (11)	0.28954 (9)	0.0179 (3)	
H34	0.5332	0.4398	0.3127	0.022*	
C35	0.57070 (12)	0.38537 (12)	0.20640 (9)	0.0193 (3)	
H35	0.5448	0.4517	0.1723	0.023*	
C36	0.61554 (13)	0.29522 (12)	0.17255 (9)	0.0201 (3)	
C37	0.65297 (12)	0.19634 (12)	0.22104 (9)	0.0197 (3)	
H37	0.6830	0.1353	0.1979	0.024*	
C38	0.58807 (15)	0.39437 (14)	0.03902 (9)	0.0278 (3)	
H38A	0.5994	0.3840	−0.0168	0.042*	
H38B	0.6362	0.4603	0.0400	0.042*	
H38C	0.5030	0.4070	0.0578	0.042*	
C39	0.87551 (14)	0.51225 (13)	0.09507 (10)	0.0265 (3)	
H39A	0.8735	0.5466	0.0374	0.040*	
H39B	0.9234	0.5611	0.1163	0.040*	
H39C	0.7935	0.5040	0.1264	0.040*	
C40	0.97262 (12)	0.20264 (12)	0.32586 (9)	0.0204 (3)	
C41	1.00957 (13)	0.16588 (12)	0.25382 (10)	0.0226 (3)	
H41	1.0455	0.0942	0.2548	0.027*	
C42	0.99184 (13)	0.23744 (12)	0.18191 (10)	0.0240 (3)	
H42	1.0165	0.2149	0.1321	0.029*	
C43	0.93764 (13)	0.34427 (12)	0.18016 (10)	0.0227 (3)	
C44	0.90086 (12)	0.38164 (12)	0.25020 (9)	0.0205 (3)	
H44	0.8648	0.4534	0.2484	0.025*	
C45	0.91865 (12)	0.30930 (11)	0.32534 (9)	0.0192 (3)	
C46	0.89379 (12)	0.31841 (12)	0.40774 (9)	0.0203 (3)	
H46	0.8577	0.3807	0.4269	0.024*	
C47	0.93202 (12)	0.21964 (12)	0.45500 (9)	0.0200 (3)	
C48	0.91909 (12)	0.17394 (12)	0.54301 (9)	0.0202 (3)	
C49	0.96108 (13)	0.36113 (11)	0.57014 (10)	0.0216 (3)	
H49	0.9899	0.4022	0.5156	0.026*	
C50	0.96235 (13)	0.40024 (11)	0.63750 (9)	0.0213 (3)	
H50	0.9916	0.4728	0.6384	0.026*	
C51	0.91153 (12)	0.31241 (11)	0.70786 (9)	0.0196 (3)	
C52	0.87999 (12)	0.22017 (11)	0.67885 (9)	0.0189 (3)	
C53	0.82490 (12)	0.12172 (12)	0.73196 (10)	0.0214 (3)	
H53	0.8014	0.0602	0.7121	0.026*	
C54	0.80601 (13)	0.11751 (12)	0.81456 (10)	0.0227 (3)	
H54	0.7683	0.0517	0.8521	0.027*	
C55	0.84108 (13)	0.20794 (12)	0.84477 (9)	0.0223 (3)	
C56	0.89355 (13)	0.30686 (12)	0.79158 (9)	0.0212 (3)	
H56	0.9164	0.3686	0.8115	0.025*	
C57	0.86969 (15)	0.26861 (13)	0.96288 (10)	0.0278 (3)	
H57A	0.8459	0.2471	1.0225	0.042*	
H57B	0.8421	0.3453	0.9419	0.042*	
H57C	0.9574	0.2673	0.9482	0.042*	
H1n	0.671 (2)	0.5735 (19)	0.4445 (14)	0.038 (6)*	
H3n	0.6380 (19)	0.4170 (18)	0.5893 (13)	0.028 (5)*	
H5n	1.0089 (19)	0.0807 (18)	0.4222 (13)	0.027 (5)*	

### Atomic displacement parameters (Å^2^)

**Table:**

	*U*^11^	*U*^22^	*U*^33^	*U*^12^	*U*^13^	*U*^23^
O1	0.0359 (6)	0.0214 (5)	0.0176 (5)	0.0048 (4)	−0.0031 (4)	−0.0048 (4)
O2	0.0308 (5)	0.0136 (5)	0.0236 (5)	0.0013 (4)	−0.0054 (4)	−0.0034 (4)
O3	0.0347 (6)	0.0282 (5)	0.0183 (5)	0.0064 (4)	−0.0064 (4)	−0.0054 (4)
O4	0.0347 (6)	0.0228 (5)	0.0152 (5)	−0.0044 (4)	−0.0024 (4)	−0.0032 (4)
O5	0.0316 (5)	0.0128 (4)	0.0210 (5)	0.0020 (4)	−0.0047 (4)	−0.0032 (4)
O6	0.0355 (6)	0.0267 (5)	0.0155 (5)	0.0019 (4)	−0.0026 (4)	−0.0053 (4)
O7	0.0359 (6)	0.0227 (5)	0.0237 (6)	0.0047 (4)	−0.0032 (4)	−0.0044 (4)
O8	0.0305 (5)	0.0130 (5)	0.0295 (6)	0.0030 (4)	−0.0019 (4)	−0.0028 (4)
O9	0.0310 (6)	0.0265 (5)	0.0225 (5)	−0.0047 (4)	−0.0003 (4)	−0.0020 (4)
N1	0.0203 (5)	0.0151 (5)	0.0213 (6)	0.0019 (4)	−0.0028 (4)	−0.0063 (4)
N2	0.0203 (5)	0.0134 (5)	0.0172 (6)	0.0025 (4)	−0.0018 (4)	−0.0032 (4)
N3	0.0220 (6)	0.0138 (5)	0.0175 (6)	0.0008 (4)	−0.0028 (4)	−0.0039 (4)
N4	0.0187 (5)	0.0137 (5)	0.0163 (6)	0.0026 (4)	−0.0022 (4)	−0.0029 (4)
N5	0.0212 (6)	0.0149 (5)	0.0268 (6)	0.0047 (4)	−0.0042 (5)	−0.0054 (5)
N6	0.0198 (6)	0.0122 (5)	0.0241 (6)	0.0012 (4)	−0.0031 (5)	−0.0023 (4)
C1	0.0313 (8)	0.0222 (7)	0.0214 (7)	0.0047 (6)	−0.0006 (6)	−0.0034 (6)
C2	0.0183 (6)	0.0150 (6)	0.0211 (7)	0.0045 (5)	−0.0036 (5)	−0.0064 (5)
C3	0.0214 (6)	0.0177 (6)	0.0250 (7)	0.0048 (5)	−0.0059 (5)	−0.0087 (5)
C4	0.0244 (7)	0.0213 (7)	0.0220 (7)	0.0070 (5)	−0.0070 (5)	−0.0102 (5)
C5	0.0230 (7)	0.0199 (6)	0.0192 (7)	0.0087 (5)	−0.0044 (5)	−0.0043 (5)
C6	0.0188 (6)	0.0165 (6)	0.0196 (7)	0.0039 (5)	−0.0026 (5)	−0.0051 (5)
C7	0.0162 (6)	0.0163 (6)	0.0203 (7)	0.0036 (5)	−0.0030 (5)	−0.0058 (5)
C8	0.0169 (6)	0.0159 (6)	0.0197 (7)	0.0037 (5)	−0.0013 (5)	−0.0052 (5)
C9	0.0171 (6)	0.0157 (6)	0.0200 (7)	0.0030 (5)	−0.0016 (5)	−0.0067 (5)
C10	0.0167 (6)	0.0147 (6)	0.0209 (7)	0.0023 (5)	−0.0028 (5)	−0.0045 (5)
C11	0.0205 (6)	0.0136 (6)	0.0203 (7)	0.0033 (5)	−0.0021 (5)	−0.0023 (5)
C12	0.0208 (6)	0.0143 (6)	0.0216 (7)	0.0021 (5)	−0.0019 (5)	−0.0036 (5)
C13	0.0161 (6)	0.0155 (6)	0.0213 (7)	0.0008 (5)	−0.0015 (5)	−0.0039 (5)
C14	0.0159 (6)	0.0161 (6)	0.0188 (6)	0.0004 (5)	−0.0022 (5)	−0.0041 (5)
C15	0.0193 (6)	0.0152 (6)	0.0227 (7)	0.0018 (5)	−0.0027 (5)	−0.0034 (5)
C16	0.0208 (6)	0.0184 (6)	0.0227 (7)	0.0030 (5)	−0.0040 (5)	−0.0011 (5)
C17	0.0196 (6)	0.0241 (7)	0.0188 (7)	0.0004 (5)	−0.0031 (5)	−0.0044 (5)
C18	0.0206 (6)	0.0189 (6)	0.0219 (7)	0.0019 (5)	−0.0027 (5)	−0.0063 (5)
C19	0.0534 (11)	0.0373 (9)	0.0236 (8)	0.0136 (8)	−0.0114 (7)	−0.0125 (7)
C20	0.0283 (7)	0.0227 (7)	0.0206 (7)	−0.0027 (6)	−0.0016 (6)	−0.0019 (5)
C21	0.0170 (6)	0.0153 (6)	0.0193 (7)	0.0034 (5)	−0.0024 (5)	−0.0051 (5)
C22	0.0211 (6)	0.0158 (6)	0.0212 (7)	0.0015 (5)	−0.0042 (5)	−0.0067 (5)
C23	0.0236 (7)	0.0205 (7)	0.0190 (7)	0.0018 (5)	−0.0044 (5)	−0.0081 (5)
C24	0.0201 (6)	0.0191 (6)	0.0160 (6)	0.0021 (5)	−0.0020 (5)	−0.0036 (5)
C25	0.0183 (6)	0.0162 (6)	0.0177 (6)	0.0016 (5)	−0.0025 (5)	−0.0041 (5)
C26	0.0158 (6)	0.0152 (6)	0.0189 (7)	0.0026 (5)	−0.0031 (5)	−0.0045 (5)
C27	0.0183 (6)	0.0140 (6)	0.0186 (6)	0.0014 (5)	−0.0030 (5)	−0.0043 (5)
C28	0.0185 (6)	0.0143 (6)	0.0174 (6)	0.0031 (5)	−0.0034 (5)	−0.0049 (5)
C29	0.0177 (6)	0.0148 (6)	0.0189 (7)	0.0024 (5)	−0.0032 (5)	−0.0039 (5)
C30	0.0227 (6)	0.0126 (6)	0.0206 (7)	0.0022 (5)	−0.0031 (5)	−0.0023 (5)
C31	0.0201 (6)	0.0143 (6)	0.0220 (7)	0.0025 (5)	−0.0018 (5)	−0.0044 (5)
C32	0.0166 (6)	0.0150 (6)	0.0202 (7)	−0.0001 (5)	−0.0005 (5)	−0.0041 (5)
C33	0.0154 (6)	0.0149 (6)	0.0178 (6)	−0.0006 (5)	−0.0004 (5)	−0.0040 (5)
C34	0.0180 (6)	0.0159 (6)	0.0187 (6)	0.0017 (5)	−0.0015 (5)	−0.0031 (5)
C35	0.0189 (6)	0.0185 (6)	0.0187 (7)	0.0005 (5)	−0.0029 (5)	−0.0014 (5)
C36	0.0197 (6)	0.0228 (7)	0.0168 (6)	−0.0028 (5)	−0.0005 (5)	−0.0048 (5)
C37	0.0189 (6)	0.0185 (6)	0.0214 (7)	0.0003 (5)	0.0008 (5)	−0.0072 (5)
C38	0.0344 (8)	0.0289 (8)	0.0187 (7)	−0.0026 (6)	−0.0061 (6)	−0.0016 (6)
C39	0.0283 (7)	0.0213 (7)	0.0274 (8)	0.0012 (6)	−0.0039 (6)	−0.0021 (6)
C40	0.0173 (6)	0.0159 (6)	0.0270 (7)	0.0009 (5)	−0.0031 (5)	−0.0039 (5)
C41	0.0210 (6)	0.0167 (6)	0.0298 (8)	0.0027 (5)	−0.0015 (6)	−0.0075 (6)
C42	0.0227 (7)	0.0210 (7)	0.0278 (8)	−0.0001 (5)	0.0000 (6)	−0.0086 (6)
C43	0.0221 (7)	0.0194 (7)	0.0249 (7)	−0.0011 (5)	−0.0022 (5)	−0.0034 (6)
C44	0.0182 (6)	0.0160 (6)	0.0263 (7)	0.0016 (5)	−0.0040 (5)	−0.0034 (5)
C45	0.0164 (6)	0.0161 (6)	0.0247 (7)	0.0010 (5)	−0.0023 (5)	−0.0046 (5)
C46	0.0180 (6)	0.0146 (6)	0.0282 (7)	0.0017 (5)	−0.0037 (5)	−0.0055 (5)
C47	0.0183 (6)	0.0157 (6)	0.0263 (7)	0.0015 (5)	−0.0032 (5)	−0.0065 (5)
C48	0.0173 (6)	0.0150 (6)	0.0276 (7)	0.0030 (5)	−0.0031 (5)	−0.0046 (5)
C49	0.0221 (7)	0.0136 (6)	0.0264 (7)	−0.0003 (5)	−0.0007 (5)	−0.0021 (5)
C50	0.0214 (6)	0.0137 (6)	0.0266 (7)	0.0002 (5)	−0.0015 (5)	−0.0025 (5)
C51	0.0163 (6)	0.0152 (6)	0.0260 (7)	0.0019 (5)	−0.0030 (5)	−0.0031 (5)
C52	0.0167 (6)	0.0144 (6)	0.0244 (7)	0.0036 (5)	−0.0030 (5)	−0.0029 (5)
C53	0.0173 (6)	0.0154 (6)	0.0302 (8)	0.0017 (5)	−0.0030 (5)	−0.0035 (5)
C54	0.0188 (6)	0.0174 (6)	0.0283 (8)	−0.0008 (5)	−0.0013 (5)	0.0000 (5)
C55	0.0192 (6)	0.0208 (7)	0.0240 (7)	0.0024 (5)	−0.0004 (5)	−0.0022 (6)
C56	0.0198 (6)	0.0169 (6)	0.0251 (7)	0.0010 (5)	−0.0018 (5)	−0.0029 (5)
C57	0.0346 (8)	0.0212 (7)	0.0261 (8)	0.0014 (6)	−0.0020 (6)	−0.0050 (6)

### Geometric parameters (Å, º)

**Table:**

O1—C5	1.3765 (18)	C20—H20A	0.9800
O1—C1	1.4240 (19)	C20—H20B	0.9800
O2—C10	1.2285 (17)	C20—H20C	0.9800
O3—C17	1.3732 (18)	C21—C22	1.403 (2)
O3—C19	1.422 (2)	C21—C26	1.4140 (19)
O4—C24	1.3721 (17)	C22—C23	1.374 (2)
O4—C20	1.4232 (18)	C22—H22	0.9500
O5—C29	1.2273 (17)	C23—C24	1.420 (2)
O6—C36	1.3732 (18)	C23—H23	0.9500
O6—C38	1.4204 (19)	C24—C25	1.378 (2)
O7—C43	1.3768 (19)	C25—C26	1.4111 (19)
O7—C39	1.4202 (18)	C25—H25	0.9500
O8—C48	1.2292 (18)	C26—C27	1.4245 (19)
O9—C55	1.3703 (19)	C27—C28	1.3854 (19)
O9—C57	1.424 (2)	C27—H27	0.9500
N1—C2	1.3649 (19)	C28—C29	1.4604 (19)
N1—C9	1.3775 (18)	C30—C31	1.351 (2)
N1—H1n	0.91 (2)	C30—H30	0.9500
N2—C10	1.3868 (18)	C31—C32	1.4426 (19)
N2—C14	1.4070 (18)	C31—H31	0.9500
N2—C11	1.4097 (17)	C32—C37	1.386 (2)
N3—C21	1.3658 (18)	C32—C33	1.4091 (18)
N3—C28	1.3751 (18)	C33—C34	1.3894 (19)
N3—H3n	0.91 (2)	C34—C35	1.396 (2)
N4—C29	1.3897 (18)	C34—H34	0.9500
N4—C30	1.4099 (16)	C35—C36	1.401 (2)
N4—C33	1.4099 (18)	C35—H35	0.9500
N5—C40	1.366 (2)	C36—C37	1.392 (2)
N5—C47	1.3805 (18)	C37—H37	0.9500
N5—H5n	0.90 (2)	C38—H38A	0.9800
N6—C48	1.3886 (19)	C38—H38B	0.9800
N6—C52	1.4096 (19)	C38—H38C	0.9800
N6—C49	1.4139 (18)	C39—H39A	0.9800
C1—H1A	0.9800	C39—H39B	0.9800
C1—H1B	0.9800	C39—H39C	0.9800
C1—H1C	0.9800	C40—C41	1.402 (2)
C2—C3	1.405 (2)	C40—C45	1.4177 (18)
C2—C7	1.4103 (19)	C41—C42	1.372 (2)
C3—C4	1.374 (2)	C41—H41	0.9500
C3—H3A	0.9500	C42—C43	1.418 (2)
C4—C5	1.415 (2)	C42—H42	0.9500
C4—H4	0.9500	C43—C44	1.375 (2)
C5—C6	1.378 (2)	C44—C45	1.420 (2)
C6—C7	1.4167 (19)	C44—H44	0.9500
C6—H6	0.9500	C45—C46	1.420 (2)
C7—C8	1.4202 (19)	C46—C47	1.3855 (19)
C8—C9	1.3884 (19)	C46—H46	0.9500
C8—H8	0.9500	C47—C48	1.459 (2)
C9—C10	1.4591 (19)	C49—C50	1.350 (2)
C11—C12	1.352 (2)	C49—H49	0.9500
C11—H11	0.9500	C50—C51	1.4432 (19)
C12—C13	1.4424 (19)	C50—H50	0.9500
C12—H12	0.9500	C51—C56	1.398 (2)
C13—C18	1.396 (2)	C51—C52	1.402 (2)
C13—C14	1.4016 (18)	C52—C53	1.3965 (19)
C14—C15	1.3961 (19)	C53—C54	1.382 (2)
C15—C16	1.384 (2)	C53—H53	0.9500
C15—H15	0.9500	C54—C55	1.409 (2)
C16—C17	1.408 (2)	C54—H54	0.9500
C16—H16	0.9500	C55—C56	1.391 (2)
C17—C18	1.385 (2)	C56—H56	0.9500
C18—H18	0.9500	C57—H57A	0.9800
C19—H19A	0.9800	C57—H57B	0.9800
C19—H19B	0.9800	C57—H57C	0.9800
C19—H19C	0.9800		
			
C5—O1—C1	116.08 (12)	C26—C25—H25	121.2
C17—O3—C19	116.73 (12)	C25—C26—C21	120.11 (13)
C24—O4—C20	116.84 (12)	C25—C26—C27	133.31 (13)
C36—O6—C38	117.51 (12)	C21—C26—C27	106.58 (12)
C43—O7—C39	116.59 (12)	C28—C27—C26	106.95 (12)
C55—O9—C57	117.19 (12)	C28—C27—H27	126.5
C2—N1—C9	108.82 (12)	C26—C27—H27	126.5
C2—N1—H1n	126.2 (14)	N3—C28—C27	109.16 (12)
C9—N1—H1n	124.9 (14)	N3—C28—C29	117.84 (12)
C10—N2—C14	125.10 (11)	C27—C28—C29	132.63 (13)
C10—N2—C11	126.08 (12)	O5—C29—N4	120.34 (13)
C14—N2—C11	107.48 (11)	O5—C29—C28	121.45 (12)
C21—N3—C28	109.04 (12)	N4—C29—C28	118.21 (11)
C21—N3—H3n	125.8 (13)	C31—C30—N4	109.85 (12)
C28—N3—H3n	125.2 (13)	C31—C30—H30	125.1
C29—N4—C30	125.87 (12)	N4—C30—H30	125.1
C29—N4—C33	124.54 (11)	C30—C31—C32	107.94 (12)
C30—N4—C33	107.51 (11)	C30—C31—H31	126.0
C40—N5—C47	109.01 (12)	C32—C31—H31	126.0
C40—N5—H5n	126.4 (13)	C37—C32—C33	120.10 (13)
C47—N5—H5n	124.6 (13)	C37—C32—C31	132.79 (13)
C48—N6—C52	125.13 (12)	C33—C32—C31	107.11 (12)
C48—N6—C49	125.52 (13)	C34—C33—C32	121.58 (13)
C52—N6—C49	107.23 (12)	C34—C33—N4	130.78 (12)
O1—C1—H1A	109.5	C32—C33—N4	107.59 (11)
O1—C1—H1B	109.5	C33—C34—C35	117.79 (12)
H1A—C1—H1B	109.5	C33—C34—H34	121.1
O1—C1—H1C	109.5	C35—C34—H34	121.1
H1A—C1—H1C	109.5	C34—C35—C36	120.85 (13)
H1B—C1—H1C	109.5	C34—C35—H35	119.6
N1—C2—C3	129.53 (13)	C36—C35—H35	119.6
N1—C2—C7	108.48 (12)	O6—C36—C37	115.04 (12)
C3—C2—C7	121.99 (13)	O6—C36—C35	124.02 (13)
C4—C3—C2	116.96 (13)	C37—C36—C35	120.94 (13)
C4—C3—H3A	121.5	C32—C37—C36	118.72 (13)
C2—C3—H3A	121.5	C32—C37—H37	120.6
C3—C4—C5	121.87 (14)	C36—C37—H37	120.6
C3—C4—H4	119.1	O6—C38—H38A	109.5
C5—C4—H4	119.1	O6—C38—H38B	109.5
O1—C5—C6	125.16 (14)	H38A—C38—H38B	109.5
O1—C5—C4	113.18 (13)	O6—C38—H38C	109.5
C6—C5—C4	121.65 (14)	H38A—C38—H38C	109.5
C5—C6—C7	117.55 (13)	H38B—C38—H38C	109.5
C5—C6—H6	121.2	O7—C39—H39A	109.5
C7—C6—H6	121.2	O7—C39—H39B	109.5
C2—C7—C6	119.98 (13)	H39A—C39—H39B	109.5
C2—C7—C8	106.72 (12)	O7—C39—H39C	109.5
C6—C7—C8	133.31 (13)	H39A—C39—H39C	109.5
C9—C8—C7	106.92 (12)	H39B—C39—H39C	109.5
C9—C8—H8	126.5	N5—C40—C41	129.92 (13)
C7—C8—H8	126.5	N5—C40—C45	108.22 (13)
N1—C9—C8	109.05 (12)	C41—C40—C45	121.86 (14)
N1—C9—C10	118.01 (12)	C42—C41—C40	117.44 (13)
C8—C9—C10	132.49 (13)	C42—C41—H41	121.3
O2—C10—N2	120.42 (13)	C40—C41—H41	121.3
O2—C10—C9	121.34 (13)	C41—C42—C43	121.54 (14)
N2—C10—C9	118.24 (12)	C41—C42—H42	119.2
C12—C11—N2	109.64 (12)	C43—C42—H42	119.2
C12—C11—H11	125.2	C44—C43—O7	125.32 (13)
N2—C11—H11	125.2	C44—C43—C42	121.79 (14)
C11—C12—C13	107.93 (12)	O7—C43—C42	112.89 (14)
C11—C12—H12	126.0	C43—C44—C45	117.73 (13)
C13—C12—H12	126.0	C43—C44—H44	121.1
C18—C13—C14	120.62 (13)	C45—C44—H44	121.1
C18—C13—C12	132.28 (13)	C40—C45—C46	106.58 (13)
C14—C13—C12	107.10 (12)	C40—C45—C44	119.64 (14)
C15—C14—C13	121.68 (13)	C46—C45—C44	133.78 (13)
C15—C14—N2	130.44 (13)	C47—C46—C45	107.27 (12)
C13—C14—N2	107.85 (11)	C47—C46—H46	126.4
C16—C15—C14	117.16 (13)	C45—C46—H46	126.4
C16—C15—H15	121.4	N5—C47—C46	108.92 (13)
C14—C15—H15	121.4	N5—C47—C48	117.63 (12)
C15—C16—C17	121.60 (13)	C46—C47—C48	132.99 (13)
C15—C16—H16	119.2	O8—C48—N6	120.42 (14)
C17—C16—H16	119.2	O8—C48—C47	121.27 (14)
O3—C17—C18	124.65 (13)	N6—C48—C47	118.31 (12)
O3—C17—C16	114.37 (13)	C50—C49—N6	109.76 (13)
C18—C17—C16	120.98 (14)	C50—C49—H49	125.1
C17—C18—C13	117.94 (13)	N6—C49—H49	125.1
C17—C18—H18	121.0	C49—C50—C51	107.98 (13)
C13—C18—H18	121.0	C49—C50—H50	126.0
O3—C19—H19A	109.5	C51—C50—H50	126.0
O3—C19—H19B	109.5	C56—C51—C52	120.89 (13)
H19A—C19—H19B	109.5	C56—C51—C50	131.99 (14)
O3—C19—H19C	109.5	C52—C51—C50	107.11 (13)
H19A—C19—H19C	109.5	C53—C52—C51	121.46 (14)
H19B—C19—H19C	109.5	C53—C52—N6	130.59 (14)
O4—C20—H20A	109.5	C51—C52—N6	107.92 (12)
O4—C20—H20B	109.5	C54—C53—C52	117.24 (14)
H20A—C20—H20B	109.5	C54—C53—H53	121.4
O4—C20—H20C	109.5	C52—C53—H53	121.4
H20A—C20—H20C	109.5	C53—C54—C55	121.91 (14)
H20B—C20—H20C	109.5	C53—C54—H54	119.0
N3—C21—C22	129.94 (13)	C55—C54—H54	119.0
N3—C21—C26	108.27 (12)	O9—C55—C56	124.50 (14)
C22—C21—C26	121.79 (13)	O9—C55—C54	114.82 (13)
C23—C22—C21	117.27 (13)	C56—C55—C54	120.67 (14)
C23—C22—H22	121.4	C55—C56—C51	117.77 (14)
C21—C22—H22	121.4	C55—C56—H56	121.1
C22—C23—C24	121.57 (13)	C51—C56—H56	121.1
C22—C23—H23	119.2	O9—C57—H57A	109.5
C24—C23—H23	119.2	O9—C57—H57B	109.5
O4—C24—C25	124.97 (13)	H57A—C57—H57B	109.5
O4—C24—C23	113.43 (12)	O9—C57—H57C	109.5
C25—C24—C23	121.59 (13)	H57A—C57—H57C	109.5
C24—C25—C26	117.66 (13)	H57B—C57—H57C	109.5
C24—C25—H25	121.2		
			
C9—N1—C2—C3	−179.26 (13)	C27—C28—C29—O5	151.80 (15)
C9—N1—C2—C7	1.13 (15)	N3—C28—C29—N4	160.58 (12)
N1—C2—C3—C4	−179.08 (13)	C27—C28—C29—N4	−27.3 (2)
C7—C2—C3—C4	0.5 (2)	C29—N4—C30—C31	−164.97 (13)
C2—C3—C4—C5	0.2 (2)	C33—N4—C30—C31	−0.85 (16)
C1—O1—C5—C6	−2.1 (2)	N4—C30—C31—C32	0.62 (16)
C1—O1—C5—C4	177.08 (12)	C30—C31—C32—C37	179.82 (15)
C3—C4—C5—O1	179.96 (12)	C30—C31—C32—C33	−0.15 (16)
C3—C4—C5—C6	−0.8 (2)	C37—C32—C33—C34	1.8 (2)
O1—C5—C6—C7	179.79 (12)	C31—C32—C33—C34	−178.19 (13)
C4—C5—C6—C7	0.7 (2)	C37—C32—C33—N4	179.66 (12)
N1—C2—C7—C6	179.02 (12)	C31—C32—C33—N4	−0.36 (15)
C3—C2—C7—C6	−0.6 (2)	C29—N4—C33—C34	−17.3 (2)
N1—C2—C7—C8	−0.76 (14)	C30—N4—C33—C34	178.28 (14)
C3—C2—C7—C8	179.59 (12)	C29—N4—C33—C32	165.11 (12)
C5—C6—C7—C2	0.04 (19)	C30—N4—C33—C32	0.73 (15)
C5—C6—C7—C8	179.74 (14)	C32—C33—C34—C35	−1.3 (2)
C2—C7—C8—C9	0.11 (14)	N4—C33—C34—C35	−178.60 (13)
C6—C7—C8—C9	−179.63 (14)	C33—C34—C35—C36	0.1 (2)
C2—N1—C9—C8	−1.07 (15)	C38—O6—C36—C37	179.90 (13)
C2—N1—C9—C10	−174.29 (11)	C38—O6—C36—C35	0.6 (2)
C7—C8—C9—N1	0.58 (15)	C34—C35—C36—O6	−179.95 (13)
C7—C8—C9—C10	172.45 (13)	C34—C35—C36—C37	0.7 (2)
C14—N2—C10—O2	12.4 (2)	C33—C32—C37—C36	−1.0 (2)
C11—N2—C10—O2	−152.69 (14)	C31—C32—C37—C36	179.04 (15)
C14—N2—C10—C9	−166.47 (12)	O6—C36—C37—C32	−179.63 (12)
C11—N2—C10—C9	28.5 (2)	C35—C36—C37—C32	−0.3 (2)
N1—C9—C10—O2	19.73 (19)	C47—N5—C40—C41	179.54 (15)
C8—C9—C10—O2	−151.56 (15)	C47—N5—C40—C45	−0.65 (16)
N1—C9—C10—N2	−161.42 (12)	N5—C40—C41—C42	179.94 (15)
C8—C9—C10—N2	27.3 (2)	C45—C40—C41—C42	0.1 (2)
C10—N2—C11—C12	167.69 (13)	C40—C41—C42—C43	−0.4 (2)
C14—N2—C11—C12	0.46 (16)	C39—O7—C43—C44	0.0 (2)
N2—C11—C12—C13	−0.26 (16)	C39—O7—C43—C42	−179.45 (13)
C11—C12—C13—C18	−179.61 (15)	C41—C42—C43—C44	0.4 (2)
C11—C12—C13—C14	−0.03 (16)	C41—C42—C43—O7	179.88 (14)
C18—C13—C14—C15	−1.9 (2)	O7—C43—C44—C45	−179.57 (13)
C12—C13—C14—C15	178.45 (13)	C42—C43—C44—C45	−0.1 (2)
C18—C13—C14—N2	179.95 (12)	N5—C40—C45—C46	0.47 (16)
C12—C13—C14—N2	0.31 (15)	C41—C40—C45—C46	−179.70 (13)
C10—N2—C14—C15	14.2 (2)	N5—C40—C45—C44	−179.74 (13)
C11—N2—C14—C15	−178.39 (14)	C41—C40—C45—C44	0.1 (2)
C10—N2—C14—C13	−167.86 (13)	C43—C44—C45—C40	−0.1 (2)
C11—N2—C14—C13	−0.47 (15)	C43—C44—C45—C46	179.62 (15)
C13—C14—C15—C16	1.5 (2)	C40—C45—C46—C47	−0.12 (16)
N2—C14—C15—C16	179.18 (14)	C44—C45—C46—C47	−179.86 (15)
C14—C15—C16—C17	0.0 (2)	C40—N5—C47—C46	0.58 (16)
C19—O3—C17—C18	1.6 (2)	C40—N5—C47—C48	173.82 (12)
C19—O3—C17—C16	−179.52 (15)	C45—C46—C47—N5	−0.27 (16)
C15—C16—C17—O3	179.88 (13)	C45—C46—C47—C48	−172.09 (15)
C15—C16—C17—C18	−1.2 (2)	C52—N6—C48—O8	−9.4 (2)
O3—C17—C18—C13	179.62 (13)	C49—N6—C48—O8	151.94 (14)
C16—C17—C18—C13	0.8 (2)	C52—N6—C48—C47	170.13 (13)
C14—C13—C18—C17	0.7 (2)	C49—N6—C48—C47	−28.6 (2)
C12—C13—C18—C17	−179.75 (15)	N5—C47—C48—O8	−21.2 (2)
C28—N3—C21—C22	179.21 (13)	C46—C47—C48—O8	150.07 (16)
C28—N3—C21—C26	−1.06 (15)	N5—C47—C48—N6	159.32 (13)
N3—C21—C22—C23	179.75 (13)	C46—C47—C48—N6	−29.4 (2)
C26—C21—C22—C23	0.1 (2)	C48—N6—C49—C50	−164.41 (13)
C21—C22—C23—C24	−0.4 (2)	C52—N6—C49—C50	−0.33 (16)
C20—O4—C24—C25	−6.1 (2)	N6—C49—C50—C51	0.33 (16)
C20—O4—C24—C23	174.51 (12)	C49—C50—C51—C56	178.62 (15)
C22—C23—C24—O4	179.66 (13)	C49—C50—C51—C52	−0.20 (16)
C22—C23—C24—C25	0.2 (2)	C56—C51—C52—C53	2.8 (2)
O4—C24—C25—C26	−179.09 (12)	C50—C51—C52—C53	−178.19 (12)
C23—C24—C25—C26	0.3 (2)	C56—C51—C52—N6	−178.98 (12)
C24—C25—C26—C21	−0.59 (19)	C50—C51—C52—N6	−0.01 (15)
C24—C25—C26—C27	179.43 (14)	C48—N6—C52—C53	−17.7 (2)
N3—C21—C26—C25	−179.31 (12)	C49—N6—C52—C53	178.16 (14)
C22—C21—C26—C25	0.4 (2)	C48—N6—C52—C51	164.35 (12)
N3—C21—C26—C27	0.67 (14)	C49—N6—C52—C51	0.20 (15)
C22—C21—C26—C27	−179.57 (12)	C51—C52—C53—C54	−1.9 (2)
C25—C26—C27—C28	179.94 (14)	N6—C52—C53—C54	−179.63 (13)
C21—C26—C27—C28	−0.04 (14)	C52—C53—C54—C55	−0.4 (2)
C21—N3—C28—C27	1.05 (15)	C57—O9—C55—C56	−12.7 (2)
C21—N3—C28—C29	174.94 (11)	C57—O9—C55—C54	168.68 (13)
C26—C27—C28—N3	−0.61 (15)	C53—C54—C55—O9	−179.51 (13)
C26—C27—C28—C29	−173.26 (13)	C53—C54—C55—C56	1.8 (2)
C30—N4—C29—O5	152.36 (14)	O9—C55—C56—C51	−179.45 (13)
C33—N4—C29—O5	−9.2 (2)	C54—C55—C56—C51	−0.9 (2)
C30—N4—C29—C28	−28.5 (2)	C52—C51—C56—C55	−1.4 (2)
C33—N4—C29—C28	169.92 (12)	C50—C51—C56—C55	179.95 (14)
N3—C28—C29—O5	−20.34 (19)		

### Hydrogen-bond geometry (Å, º)

**Table:**

*D*—H···*A*	*D*—H	H···*A*	*D*···*A*	*D*—H···*A*
N1—H1*n*···O5	0.91 (2)	1.98 (2)	2.8536 (16)	159 (2)
N3—H3*n*···O2	0.91 (2)	1.97 (2)	2.8384 (16)	159.0 (19)
N5—H5*n*···O8^i^	0.90 (2)	2.00 (2)	2.8796 (15)	163.9 (19)

Symmetry code: (i) −*x*+2, −*y*, −*z*+1.
